# A Novel Inverse Solution of Contact Force Based on a Sparse Tactile Sensor Array

**DOI:** 10.3390/s18020351

**Published:** 2018-01-26

**Authors:** Weiting Liu, Chunxin Gu, Ruimin Zeng, Ping Yu, Xin Fu

**Affiliations:** The State Key Laboratory of Fluid Power & Mechatronic Systems, Department of Mechanical Engineering, Zhejiang University, Hangzhou 310027, Zhejiang, China; liuwt@zju.edu.cn (W.L.); zrm@zju.edu.cn (R.Z.); yuping55@zju.edu.cn (P.Y.); xfu@zju.edu.cn (X.F.)

**Keywords:** sparse tactile sensor array, inverse problem, correlation coefficient, elastomer cover

## Abstract

High-density tactile sensing has been pursued for humanoid robotic hands to obtain contact force information while the elastomer skin cover is traditionally considered to impair the force discrimination. In this work, we try to utilize the diffusion effect of the elastomer cover to identify an arbitrary contact force load just based on a sparse tactile sensor array. By numerical analysis, we proved the monotonous relation between the Pearson’s correlation coefficient and the relative distance of two single-force loads. Then, we meshed the elastomer surface and conducted the calibration load process to establish the calibration database of the sensing outputs. Afterwards, we applied the correlation method to the database and the sensing output of the unknown load to determine its location and intensity. For validation tests of the proposed method, we designed and fabricated a 3 × 3 sparse tactile sensor array with flat elastomer cover and established an automatic three-axis loading system. The validation tests were implemented including 100 random points with force intensity ranging from 0.1 to 1 N. The test results show that the method has good accuracy of detecting force load with the mean location error of 0.46 mm and the mean intensity error of 0.043 N, which meets the basic requirements of tactile sensing. Therefore, it is feasible for the sparse tactile sensor array to realize high-density load detection.

## 1. Introduction

Tactile sensing is indispensable for both human hand and humanoid robotic hands to grasp or manipulate objects as it offers the feedback information about the force load location and intensity. For human hand, the density of mechanoreceptors for tactile sensing is very high, about 17,000 units in a hand, leading to an excellent spatial resolution of about 1.6 mm for force detection [1]. However, it is extremely difficult for replicating such a high density of tactile sensor units in a humanoid robotic hand with existing technologies. On one hand, the space of humanoid robotic hands is not large enough for the existing tactile sensor units, which greatly limits the space arrangement of sensor arrays. On the other hand, a high-density tactile sensor array will bring large numbers of wire connection issues and a burden of large signal processing works, which adds to the complexity of integration into artificial hands. There have been many tactile sensor arrays developed with different sensing mechanism employed, such as piezoresistive [2,3,4], piezoelectric [5,6,7], capacitive [8,9,10], and optical systems [11,12,13,14], etc. In these tactile sensor arrays, the sensing surface is divided into scattered areas without a continuous elastomer cover and the sensor units are usually independent with each other so that each sensor unit is like a pixel of an image. Herein, the spatial resolution of force identification depends on the density of the sensor array or the spacing of adjacent units. Due to the space gap between the adjacent units, there remains blind areas for force detection, which leads to an inability of detecting an arbitrary load. Most of these past studies have focused on realizing a high-density sensor array to achieve high spatial resolution, but ignore the possibility of force identification just based on a relatively sparse tactile sensor array. Additionally, some other studies have concentrated on grasp control for specific tasks by estimating the contact force components and paid less attention to the requirements of high spatial resolution [15,16,17].

In fact, the elastomer skin cover is necessary for artificial hands to have a humanoid appearance, protect tactile sensors from possible damage or dirt, and maintain stable contact or grasp. However, for those tactile sensor arrays covered with a continuous elastomer layer, the elastomer cover is usually viewed with bias in terms of mechanical filtering effects impairing spatial discrimination. Shimojo [18] first analyzed the mechanical filtering effect of elastic cover for tactile sensor using the finite element method and concluded that the cover greatly decreases the sensor’s spatial resolution. Cabibihan et al. [19] investigated various thicknesses of synthetic skin and found that it blurs the mechanical signals transmitted to the embedded sensor. Actually, from the perspective of signal distribution, the elastomer layer offers a diffusion effect to mechanical stimulus because a load on any location of the elastomer surface will impact nearby sensor units below, which inspires sparse sensor array to gain the capacity of high spatial resolution through the inverse problem of the input-output sensing system.

Briefly, the inverse problem of the tactile sensing system is that given the output of the sensor data, the force distribution on the elastomer surface is derived. A few studies have paid attention to the problem and the proposed solutions mainly consist of two methods: one is the analytical calculation method and the other is the machine learning method. The previous analytical calculation methods [20,21] are mainly based on the analytical model of strain or stress distribution in an elastic half-space to retrieve point load. By discretization of the inverse problem, some researchers have considered force loads with area distribution. Seminara et al. [22] developed an algorithm based on the stress distribution model for estimating the contact forces distribution and tested it on simulated single normal contact data. However, the results of the analytical methods largely depend on the accuracy of the analytical model that is usually based on some ideal hypothesis, but is actually influenced by the practical material property and fabrication process. Machine learning algorithms can be used for the classification of features of the contact surfaces, e.g., textures [23,24,25] and contact patterns [26,27,28]. Machine learning techniques are appealing whenever complex mechanisms characterize the input–output relationship but require a large amount of sample data and specific empirical induction for a specific task, which still encourages high-density tactile sensor arrays and limits their widespread application in practical usage.

In this work, for a sparse tactile sensor array, we proposed a novel inverse solution of contact force utilizing the diffusion effect of the elastomer cover based on the analytical calculation model and the data from the calibration experiments. With respect to the past studies, the novelty of this work is that it is effective to predict an arbitrary single force load just by the sparse tactile sensor array and the easy-to-implement inverse solution. The method adopts Pearson’s correlation coefficient to formalize the impact of the load location on the sensing output relation. Combining the stress distribution model under the point load and the numerical analysis method, we tried to prove the feasibility of the inverse method. Then, for the validation tests, we developed a 3 × 3 sparse tactile sensor array and an automatic three-axis loading set-up. Afterwards, we conducted the experiments and obtained the calibration database and the output data of 100 random point loads to validate the proposed method. Encouraged by the highest spatial resolution of a human fingertip as 1.6 mm and the normal manipulation force sensitivity larger than 15 g weight (0.147 N) [29], we set the reference values of prediction errors as 1 mm and 0.1 N for validation tests.

## 2. Methods

### 2.1. Description of the Method

The method exploits the diffusion effects resulting from the elastomer layer so that contact signals can be obtained together by nearby sparse sensors. In this way, each sensor unit is provided with a wider receptive field compared with those lacking the cover of the elastomer layer. Thus, the sensing outputs processed by the elastomer cover contain contact information fused with direction, location, and intensity of the force load, which indicates a possible method to determine an unknown force load by utilizing the characteristics of the sensing output. Herein, we attempt to verify the method starting with a simple situation to ignore the influence of the force direction, such that only a normal point load is considered. For a sensor unit, there seem two obvious properties related to elastomer cover: the sensing output grows monotonously (1) with the decrease of the relative distance from force load; and (2) with the increase of the force intensity. A natural idea is that the force loads on adjacent points will lead to similar outputs of the sensor array.

**Figure 1:**
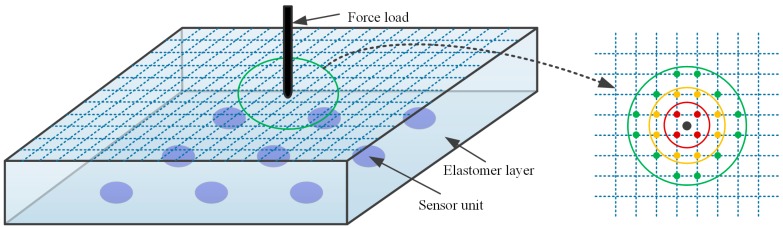
Schematic of a sensing system with mesh grids on the surface of the elastomer cover. The inset displays the load point location in the plane that the black point represents the load point and the colorful points represent the mesh points with different distances to the load point.

Based on the sparse sensor array, combined with the continuous elastomer layer, the method to determine both the location and intensity of an unknown single point force is described in the steps below:

Step 1: as shown in Figure 1, the surface of the elastomer layer is meshed and divided into scattered areas. Thus, an arbitrary force load on the surface will be surrounded with mesh points nearby.
Step 2: load on all mesh points one by one by a single normal force with unit intensity to establish a calibration database containing the respective sensing outputs. This step is called the calibration process, these force loads are called calibration loads, and the mesh points are called calibration points.
Step 3: compare the sensing output data caused by the unknown load with the calibration database to rank the calibration points by measuring the similarity of the sensing outputs and then determine the nearby points of the unknown load. Herein, Pearson’s correlation coefficient is adopted to measure the similarity of the sensing outputs. The calibration point with the largest coefficient is regarded as the closest to the unknown load so that the location of the point is used for the unknown load and called the result point.
Step 4: determine the closest sensor unit to the result point and calculate the ratio between the sensing output of the unknown load and the calibration data of the result point from that sensor unit to obtain the intensity of the unknown load with the given intensity of the calibration load.

### 2.2. Principle of the Method

In this work, we try to understand the proposed method theoretically in two aspects that will influence the location results: (1) the sensor array distribution including sensor number and sensor spacing; and (2) the relative location between the unknown load point and the calibration point.

Normally, the tactile stimuli applied on the surface of the elastomer cover are assumed to be received by the sensors as a pressure on their upper side (normal stress component in the vertical direction). Thus, we adopt the normal stress inside the elastomer layer to substitute for the sensing output. Additionally, the elastomer material, like the widely used polydimethylsiloxane (PDMS), has a linear elastic behavior when the strain is not large (40% for PDMS) [30]. To simplify the analysis, the elastomer cover is treated as an elastic half-space system regardless of the boundary effect. According to contact mechanics [31], in an elastic half-space, the normal stress *σ* in the *z* direction of a random point inside produced by a concentrated point force acting normally on the surface is expressed by the following equation:

(1) $$\sigma =-\frac{3F}{2\pi }\frac{z^{3}}{{\left(\rho^{2}+z^{2}\right)}^{5/2}}$$

where *F* is the intensity of the force load; *z* is the vertical distance from the inside point to the elastic surface; and *ρ* is the horizontal distance from the inside point to the load point. From a practical view, the value of *z* represents the depth of sensor array underneath the elastomer surface, which is usually set as a constant value. Therefore, we select *z* as a reference value and set *ρ* = *kz* and *k* as a ratio parameter so that Equation (1) can be simplified into:

(2) $$\sigma =-\frac{3F}{2\pi z^{2}}\frac{1}{{\left(k^{2}+1\right)}^{5/2}}$$

**Figure 2:**
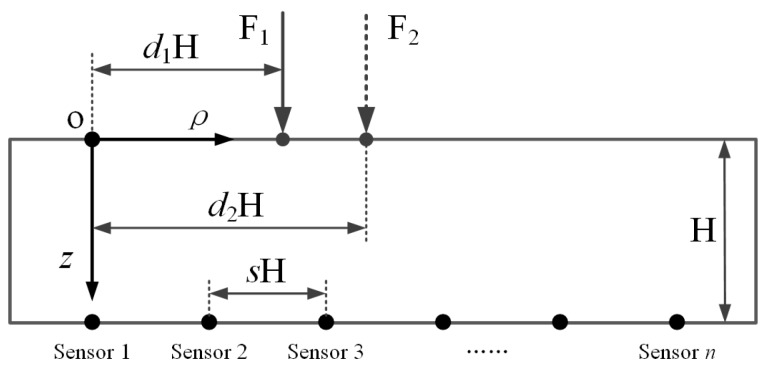
Schematic of loading on an elastomer layer with embedded sparse sensor array.

Apparently, the above equations are based on an assumption that the coordinate origin is set at the load point, which demands respective coordinate systems for different load points. To analyze the relationship between the sensing outputs from different force loads more easily, a common coordinate system is required. In Figure 2, a schematic of point loading on an elastomer layer with a distributed sensor array underneath is illustrated in a two-dimensional space. The origin of the common coordinate system is set at the point of the surface vertically above the first sensor unit. The sensor units are located at the same depth as H under the elastomer surface. The horizontal spacing between adjacent units is *s*H and the location of the force load is *d*H, in which both *s* and *d* are the ratio parameters.

For a single force load, the sensing output intensity of a sensor unit depends on the distance from the force load, and sensing output changes will be obtained from a distributed sensor array. It is naturally believed that force loads on two locations with close distance will generate similar sensing output signals and the closer the two loads, the more similar or correlated the output will be. To measure the similarity or correlation level of the two datasets, Pearson’s correlation coefficient is adopted, whose value is between −1 and 1. A higher value means a more positive linear correlation between the two datasets. For two variables X = [*x*_1_,…,*x_n_*] and Y = [*y*_1_,…,*y_n_*], both containing *n* values, the formula for Pearson’s correlation coefficient *r* is:

(3) $$r=f\left(x_{i},y_{i}\right)=\frac{\sum_{i=1}^{n}\left(x_{i}-\bar{x}\right)\left(y_{i}-\bar{y}\right)}{\sqrt{\sum_{i=1}^{n}{\left(x_{i}-\bar{x}\right)}^{2}}\sqrt{\sum_{i=1}^{n}{\left(y_{i}-\bar{y}\right)}^{2}}}$$

where $\bar{x}$ and $\bar{y}$ represent the mean values of the variables X and Y, respectively.

**Figure 3:**
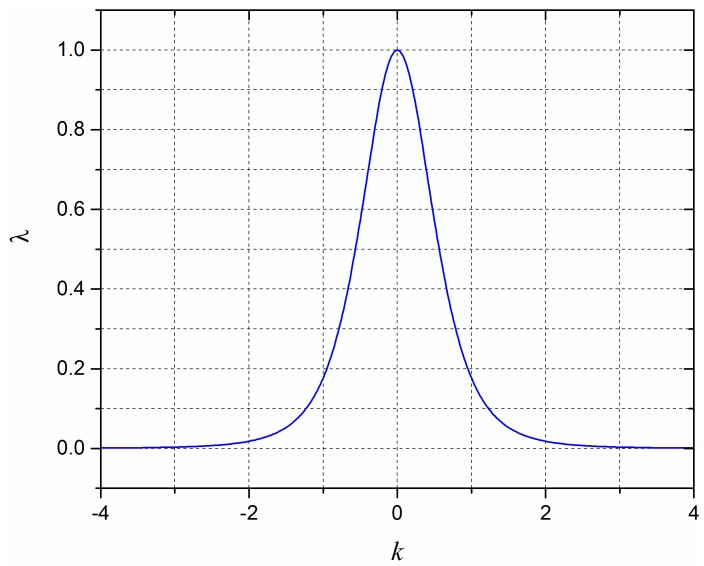
The relation curve between *λ* and *k*.

For the sensing system in Figure 2, each force load leads to corresponding sensing outputs of the sensor array, which forms a dataset with *n* values applied to Equation (3). According to the characteristics of the correlation coefficient formula, if there is a new variable X’ = *α*[*x*_1_,…,*x_n_*] modified by the linear coefficient *α* to replace X, it is obvious that the new correlation coefficient value *r’* will be the same as *r*. Additionally, from Equation (2), the force intensity variation leads to a same change ratio to each sensing output in a liner elastic system, which indicates that the force intensity will not impact the correlation level between two sensing output variables, and only the location of the force load will. Hence, to simplify the calculation of *r* in Equation (3), Equation (2) is equivalent to the following equation by ignoring the attached linear coefficients:

(4) $$\lambda ={\left(k^{2}+1\right)}^{-5/2}$$

where *λ* is denoted as a feature variable extracted from *σ* in Equation (2). The relation curve between *λ* and *k* is drawn in Figure 3, which shows that the value of *λ* decreases monotonously with the increase of the absolute value of *k*. Besides, the value of *λ* approaches to zero when the absolute value of *k* is greater than 2, which means that the effective receptive field of the sensor unit is 2H and offers a reference range for the following numerical analysis.

From Figure 2, the ratio parameters for the coordinate in the *ρ* direction of the sensor array constitute a constant dataset [0, *s*, 2*s*,…, (*n*‒1)*s*]. If the location of a force load F_j_ is *d*H, the *i*-th element in the output dataset is:

(5) $$\lambda_{ij}={\left({\left(\left(i-1\right)s-d\right)}^{2}+1\right)}^{-5/2}$$

Afterwards, for two force loads F_1_ and F_2_, with respective *d* as *d*_1_ and *d*_2_, the correlation coefficient is $r=f\left(\lambda_{i1},\lambda_{i2}\right)$. Thus, the value of *r* is related to four parameters: *n*, *s*, *d*_1_, and *d*_2_, which needs further analysis to determine the relationship. 

An ideal condition is that it maintains a monotonous correspondence between the correlation value and the relative distance of force loads as sparse as the sensor distribution could be, which will enable the discrimination of force loads at different locations. Though it is convenient to calculate the correlation coefficient *r* for any pair of output datasets with given values, it is difficult to analyze the properties by analytic deduction as Equation (3) is complicated after the replacement of variable values with Equation (5). Nevertheless, it is feasible to utilize numerical methods to analyze the value change of *r* related to the sensor array distribution and force load location.

**Figure 4:**
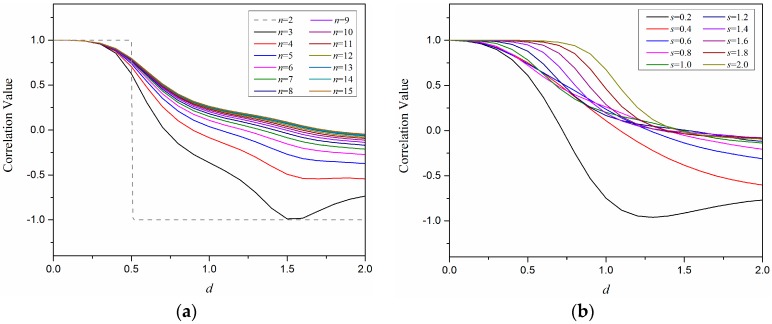
The relationships between correlation values and relative distances of two force loads with different sensor array distributions in terms of: (**a**) the number of sensor units from 2 to 15; and (**b**) the spacing parameter of sensor units from 0.2 to 2.

On one hand, in Figure 4, the relationships between the correlation value and the relative distance of F_1_ and F_2_ are illustrated by considering the sensor number *n* and the spacing parameter *s*. Herein, the location of F_1_ is fixed on the origin point and the location of F_2_ changes from 0 to 2H, as indicated in Figure 3. In Figure 4a, *n* varies among [2, 3, …, 15] while *s* is fixed at 1; in Figure 4b, *s* varies among [0.2, 0.4, …, 2.0] while *n* is fixed at 9. The results from Figure 4a show that the larger number of sensor units will increase the smoothness of the correlation value change, but the relation curves are very close when the number of sensor units is larger than nine. Additionally, it tends to have a flat, or even non-monotonous, change at the large relative distance (*d* > 1.5) when the sensor number is less than five. Especially when the sensor number is two, the correlation value has a step change and loses the monotony in the whole range. Figure 4b shows that a larger spacing (especially *s* > 1.0) will lead to a larger flat change range of the correlation value at the small relative distance. When the spacing parameter *s* is 0.2, the correlation value appears as a non-monotonous change. Hence, to guarantee a reliable identification of force load, the sensor number *n* is no less than five and the sensor spacing parameter *s* is no larger than 1.0.

**Figure 5:**
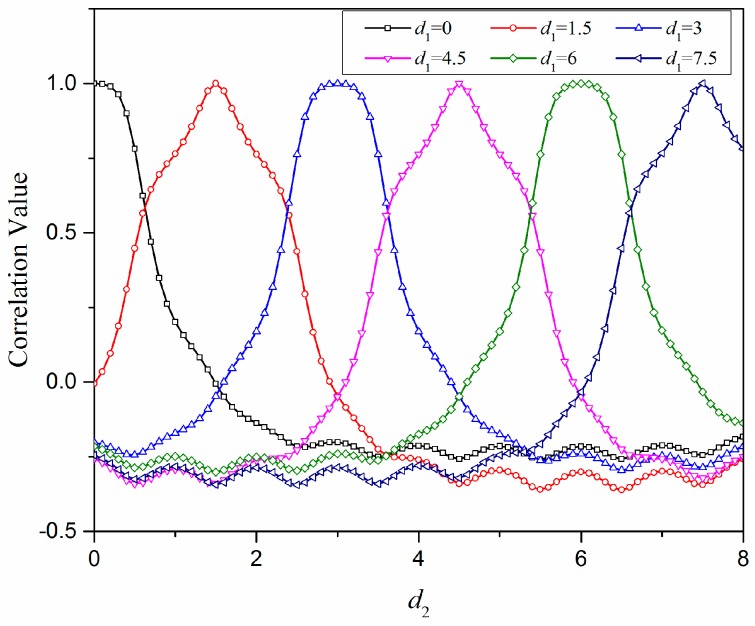
The relationships between the correlation values and the relative distances of two force loads both with variable locations when the sensor number *n* is set as 9 and the spacing parameter *s* is set as 1.0.

On the other hand, in Figure 5, the curves about the relationship between the correlation values and the relative distances under different locations are illustrated, while the sensor number *n* is 9 and the spacing parameter *s* is 1.0. The load F_1_ is set at different locations with discrete *d*_1_ varying among [0, 1.5, 3, 4.5, 6, 7.5], respectively, while the load F_2_ is set at the locations with *d*_2_ from 0 to 8. The results show that the correlation value decreases monotonously with the increase of the relative distance of the two force loads wherever the two loads are in the range of sensor units as long as the relative distance is not larger than 2H.

After that, the intensity of the unknown force load is determined according to Step 4 described in Section 2.1. The closest sensor unit to the obtained load location is determined because the sensing output attenuates with the increase of the relative distance as indicated from Figure 3, which means the sensing output from the closest sensor unit is relatively more reliable considering the practical environmental noise. Thus, if the closest sensor unit number is *i*, the calibration data of the sensor unit for the result point is *x_i_* and the sensing output of the sensor unit for the force load is *y_i_*. Since the calibration data is obtained from the unit force load in Step 3, the intensity of the fore load *F* is simply determined by calculating the ratio between *y_i_* and *x_i_* as follows:

(6) $$F=y_{i}/x_{i}$$

Therefore, even for a sparse sensor array, it is feasible to determine both approximate location and intensity for an unknown force load by the correlation method and the established calibration database. 

### 2.3. Sparse Sensor Array Design

To verify the inverse method mentioned above, we developed a sparse tactile sensor array coated with an elastomer layer. Considering the above analysis and practical fabrication, the sensor number is selected as 9 and the thickness of the elastomer cover is 5 mm, while the spacing of adjacent sensor units is 4.5 mm so that the spacing parameter *s* here is 0.9.

**Figure 6:**
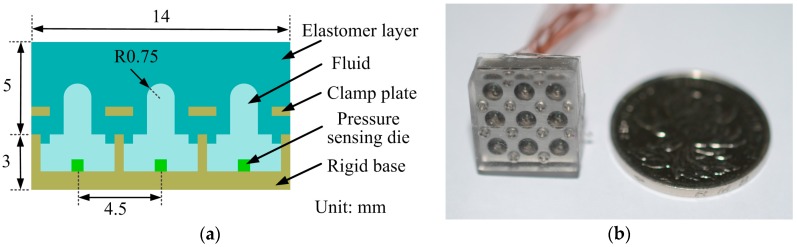
The sparse tactile sensor array with elastomer layer: (**a**) cross-section view of the sensor array; and (**b**) prototype of the sensor array.

The mechanical structure of the sensor array refers to the design introduced in our previous work [32] and only modifies the original separate elastomer bumps by covering another PDMS layer with a flat surface. As shown in Figure 6a, the sensor unit consists of five parts: the rigid base, the silicon die, the silicone fluid, the clamp plate, and the elastomer layer. The sensor array is composed of 3 × 3 sensor units with overall dimensions of 14 mm × 14 mm × 8 mm. In each sensor unit, the silicone fluid acts as not only a deformable layer, but also as a pressure transmission media, which, at the same time, protects the fragile silicon die and wire connections from direct solid contact to avoid possible damage. When a force is loaded on the surface of the elastomer cover, the deformation of the soft layer leads to signal changes of the adjacent sensor units. The prototype of the tactile sensor array is shown in Figure 6b.

## 3. Results

### 3.1. Test Set-Up

**Figure 7:**
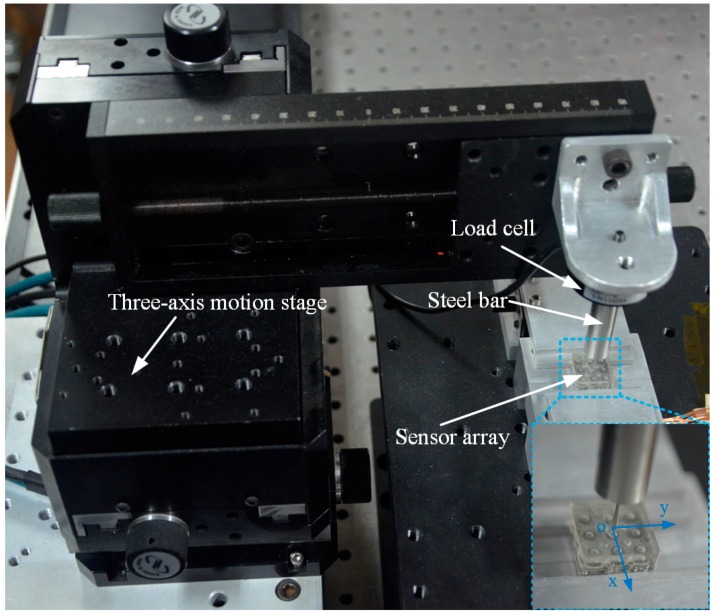
Experimental set-up for loading tests. The inset displays the steel bar loading on the surface of the sensor array.

To implement verification experiments for the inverse algorithm, the test set-up shown in Figure 7 is established. The sensor array is fixed well to a platform with the rigid base carefully clamped by a specially-designed holder. A steel loading bar with a circular bottom surface and a diameter of 1 mm is mounted onto a load cell (LSZ-F02C, OBTE, Suzhou, China) which is linked to a three-axis motion stage (VP25-XL, Newport, WA, USA) through steel accessories. The diameter of the loading bar is small enough that the produced load can be thought of as a point load. The three-axis motion stage can be driven automatically by program control with a motion precision of 1 μm in each direction. Additionally, by monitoring the force feedback from the load cell, the motion control system can control the load force intensity with a resolution of 0.05 N. Therefore, the steel bar can be loaded on any location of the surface of the elastomer layer with certain force intensity.

The tactile sensor array contains nine signal channels of voltage output which are processed by signal conditioning circuits with an amplification factor of 400. Both the processed signals of the sensor array and the signal of the load cell are independently acquired by a data acquisition card (USB-6343, National Instruments, Austin, TX, USA). 

### 3.2. Indentation Tests

Based on the experimental set-up, indentation tests are conducted to measure the basic sensor characteristics of the sensor array, establish the calibration database, and obtain test data for algorithm verification.

**Figure 8:**
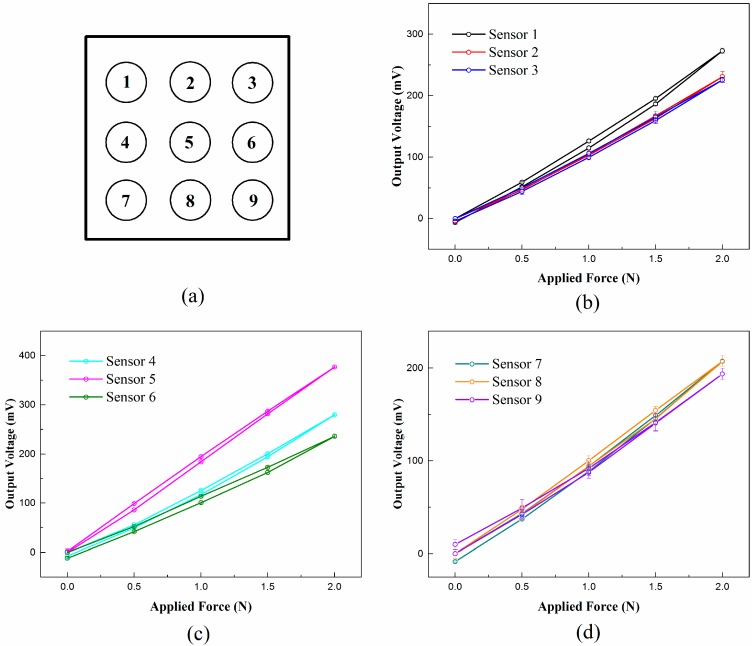
Relationships between the output voltage and the applied force for each sensor unit: (**a**) The schematic of the locations of the numbered sensor units in the sensor array; and the test results for the sensor: (**b**) 1–3; (**c**) 4–6; and (**d**) 7–9.

As shown in Figure 7, the origin point of the motion coordinate system is set at the center of the elastomer surface of the tactile sensor array while the *x*-axis and *y*-axis are parallel to the orthogonal surface edges, respectively. As the diameter of the loading bar is 1 mm, to guarantee the stability of the loading process, the loading area is restricted in the range of −6 mm to 6 mm for both *x* and *y* directions. For each loading, the coordinate values and force values have been input into the motion control system in advance. In the loading process, both output data of the sensor array and the load cell are recorded simultaneously. The experiments are divided into three parts as follows:

First, as the basic sensing characteristics, such as repeatability, hysteresis, and full-scale range are important for the accuracy of the algorithm validation, we implement the experiments, including loading and unloading processes, to analyze those characteristics for each sensor unit. Actually, the loading location will impact the output of the sensor unit, which will be complex for the tests. Thus, to simplify the measurement, we set the loading location at the surface center of each sensor unit. For each loading process, the load force increases by 0.5 N at each step, while the load force decreases by 0.5 N at each step for the unloading process. When the load force increases to 2.0 N, the indentation depth is about 1.5 mm which has been prone to damage the elastomer surface of the sensor units by observation because the diameter of the used loading bar is small. To protect the sensors, the largest force load is set as 2.0 N. For each sensor unit, the tests are repeated five times with a time interval of five minutes between each test. The test results are depicted in Figure 8, which shows that the sensor units have good linearity, good repeatability, and low hysteresis. The largest repeatability error of all sensor units is 4.89% and the largest hysteresis error is 5.53%, which offers a reliable sensing base for further calibration tests. The detailed characteristic results of each sensor are shown in Table 1.

Second, the calibration experiments are carried out with a normal load force of 1 N and a spacing of 0.5 mm between adjacent load points. Thus, the total number of the calibration points is 625 and the sensing output signals from both the load cell and the sensor array are acquired and recorded. Afterwards, a 9 × 625 calibration matrix is obtained that each column corresponds to a calibration point and each row corresponds to a sensor unit in sequence, respectively.

Third, for the test experiments, we select 100 loading points with random coordinates in the restricted area and random force intensity in the range of 0.1 to 1 N. The loading process is the same as that in the calibration experiments. The obtained test data, including 100 columns of sensing outputs, are recorded for the inverse algorithm.

### 3.3. Algorithm Tests Results

The inverse algorithm described above is realized using MATLAB 2013 and verified based on the obtained calibration data and test data.

**Figure 9:**
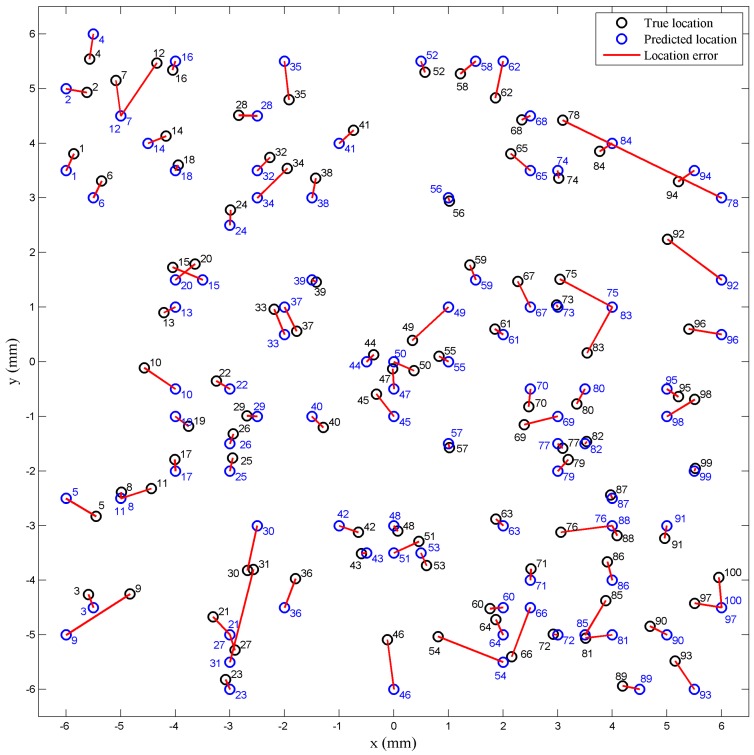
The distribution results of both true locations and predicted locations for 100 random points depicted in a two-dimensional space.

**Figure 10:**
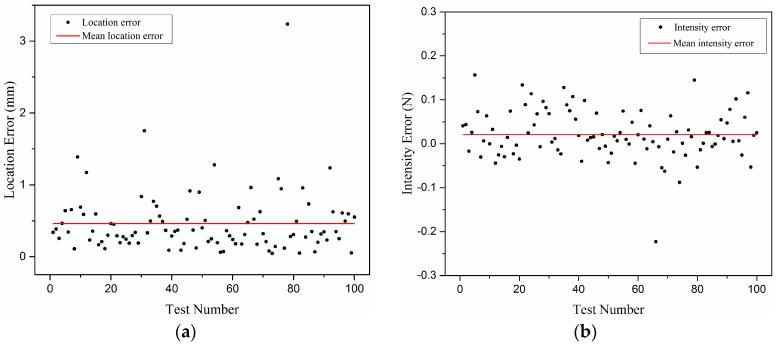
The test results of the inverse algorithm: (**a**) location error distribution; and (**b**) intensity error distribution.

The true location distributions of 100 random points and the predicted ones are depicted as black circles and blue circles, respectively, in Figure 9, while each black circle and its corresponding blue circle are connected by a red line representing the prediction error of position. Every pair of true location and predicted location is numbered nearby. The location error distributions for the 100 random points are shown in Figure 10a, revealing that the mean location error is 0.46 mm and 93% of the total points are predicted with a location error less than 1 mm. 

After the locations are determined, the prediction for the load intensity is made following the instruction of Step 4 and Equation (6) described in Section 2. The intensity error distributions are depicted in Figure 10b, which shows that 91% of the total points have an intensity error less than 0.1 N and the mean intensity error is 0.043 N.

The points with both location error less than 1 mm and intensity error less than 0.1 N account for 84%. Meanwhile, there exist some singular points with relatively large errors of location or intensity, which are mainly distributed near the boundaries of the elastomer cover. Indicated from Figure 3, it is because these areas are far from most of the sensor units, which makes the respective sensing outputs less reliable and the calibration data prone to having larger errors. However, the test results have generally met the basic requirements of tactile sensing.

## 4. Discussion and Conclusions

Based on sparse tactile sensor array covered with an elastomer layer, this paper presents a novel inverse solution using the correlation method to predict the position and intensity of an unknown single-force load. Different from the past negative views of elastomer covers for their filtering effect on mechanical signals, this work utilizes its signal diffusion effect to make mechanical stimulus detectable by a sparse sensor array. As a continuous layer, the stress distribution inside the elastomer layer has a reliable and certain analytical model supposing the elastic half-space. To understand the sensing system with a sparse sensor array, we applied the analytical model of stress distribution into a 2D space and extracted three kinds of parameters *n*, *s*, and *d* related to sensor number, sensor spacing, and load locations, respectively. Then, we analyzed the relation curves between the correlation values *r* and the relative distances *d* of any two loads regarding influences from the above three parameters. The results of the numerical analysis indicate that the sensing system will be more effective if the sensor number *n* is not less than 5 and the spacing parameter *s* is not larger than 1.0, considering the slope character of the *r*-*d* curves. Additionally, the *r*-*d* curves always maintain a monotonous change state wherever the loading locations are, which means that the correlation coefficient is effective to formalize the direct relation between the load location and the sensing outputs and, therefore, available to predict the unknown force load.

Afterwards, for validation of the proposed method, we developed a sparse tactile sensor array with total dimensions of 14 mm × 14 mm × 8 mm with a cell spacing of 4.5 mm, and set up an automatic loading system to conduct the experiments. We conducted the loading experiments to obtain the basic characteristics of each sensor unit, which shows a low repeatability error no larger than 4.89%, and a low hysteresis error no larger than 5.53% for all sensor units ranging from 0 to 2.0 N. Then, we implemented the calibration experiments to establish the calibration database and the test experiments to obtain the sensing output data of 100 point loads with random locations on the elastomer cover surface. The test results show that the proposed method behaves well to predict both the location and intensity of the unknown point load, and that the mean prediction errors are 0.46 mm for the location and 0.043 N for the intensity, which meets the basic requirements of tactile sensing.

In this work, only a normal force load is considered for the application of the proposed method. However, for a 3D force load, the tactile sensor unit will be required to have the capability of three-axis force measurement. In fact, a common method of extending the capability to three-axis force measurement is to form a new sensor cell with the symmetrical structure containing four independent sensor units, which is also feasible for the sensor array utilized in this work. After the update of the sensor unit with three-axis force detection ability, it is natural for the sensor array to measure the shear components of the force load applied to the surface of the elastomer cover. By calibration experiments for the shear force load and treating the shear components just as the normal one, it is also feasible for the application of the proposed method into the shear force case and to identify the location, direction, and intensity of the force load.

In future works, we will modify the proposed method for a three-axis load and extend its application into cases with more complicated contact patterns, such as multi-point loads.

## Figures and Tables

**Table 1:** Repeatability error and hysteresis error of the sensor units.

Sensor Number	Repeatability Error for Loading (%)	Repeatability Error for Unloading (%)	Hysteresis Error (%)
1	1.60	1.07	4.11
2	3.74	2.78	2.29
3	2.19	2.32	2.88
4	0.83	1.97	3.08
5	0.51	1.44	3.39
6	1.02	1.24	5.53
7	0.71	0.39	3.99
8	2.84	3.23	4.44
9	3.97	4.89	5.19
